# Participatory design and evaluation of digital coaching for improving health—the star multicomponent lifestyle intervention

**DOI:** 10.3389/fdgth.2025.1600535

**Published:** 2025-06-03

**Authors:** Helena Lindgren, Kaan Kilic

**Affiliations:** Department of Computing Science, Umeå University, Umeå, Sweden

**Keywords:** behaviour change, human–AI interaction, participatory design, computational argumentation, cardiovascular disease, behaviour change techniques, personalisation, mHealth

## Abstract

**Introduction:**

There are particular challenges when designing and developing a digital coaching application aimed at providing person-tailored support for lifestyle changes in multiple domains to promote health. This study explored how a participatory design process addresses challenges that materialised in a multicomponent lifestyle intervention, providing an understanding of the onboarding experience and early user engagement.

**Method:**

A participatory design methodology was applied involving a multidisciplinary team of 12 domain experts and different groups of end users in design cycles, model construction, prototyping, and evaluation. The process followed a design methodology for argument-based health information systems and a framework for layered interactive adaptive systems to engage domain experts in the development of aspects relating to the interactivity of the system. A qualitative user study was conducted with eight participants, five regular users and three nurses, focussing on the onboarding phase.

**Results:**

Contributions of this article are (i) the StarCoach, the person-tailored health-promotion intervention for multiple health behaviours supporting short and long-term goals; (ii) a framework for studying multicomponent lifestyle interventions with multiple behaviour change techniques (BCTs); and (iii) qualitative results regarding usage, adherence to, and perceived effects of the intervention with a focus on the initial phase of using the application. The five regular participants reported increased health-promoting activities during the onboarding phase and were using already habituated activities to establish a routine to use the intervention.

**Conclusion:**

The participatory design led to StarCoach embedding clusters of BCTs, which build a framework for research on multicomponent lifestyle interventions. Whether using already habituated activities to establish a routine to use the intervention could be a strategy to increase adherence and engagement in the onboarding phase and beyond will be a focus in future studies. The participants also showed increased engagement in their chosen lifestyle-change activities during the study period. The findings will be followed up in future studies to evaluate the effects on behaviour over a longer period of time.

## 1 Introduction

Targeting multiple domains in lifestyle intervention has proven successful, leading to a reduction in cardiovascular complications and improved cognition (1, 2). Multiple health behaviour change (MHBC) interventions were introduced by Prochaska et al. (3), among others, to target multiple unhealthy behaviours simultaneously. The rationale underpinning MHBCs is that unhealthy behaviours are concurrent with other unhealthy behaviours, and thus, a positive change in one would lead to a positive change in another. In addition, studies have employed computer-based systems for tailored feedback pre-MHBC interventions, which have led to positive outcomes and adherence in the long term (4).

To support individuals in changing their habits towards healthier lifestyles, a range of behaviour change systems (BCSs) have been developed following certain design principles (5). They can be seen as a subset of so-called *persuasive systems*, commonly encountered as *recommender systems* targeting various purposes (6). The design principles for BCSs include that the system should be transparent and adhere to and comply with the user’s wish for changing behaviour, aligning with the responsible design of systems based on artificial intelligence (AI) techniques (7).

Behaviour change techniques (BCTs) such as goal setting or feedback have been identified and embedded in design frameworks (5, 8). More recently, they have been organised in relation to their impact and targets, or *mechanisms of action* (MoAs), e.g., motivation or readiness to change, which in turn impact the change in a specific behaviour (9). A BCT is often combined with other BCTs to form interventions that promote healthy behaviour change in the individual.

A vast majority of lifestyle intervention applications target one particular lifestyle domain, e.g., 92% in a recent review, with the remaining 8% of applications providing a multiple-component lifestyle intervention (10). When combining different lifestyle domains, the design of the intervention must take the differences into account, as certain BCTs may have a positive impact on adherence in one domain while having a negative impact in another domain (11). The complexity and ethical aspects of multicomponent lifestyle interventions call for an inclusive and interdisciplinary design process when developing systems promoting behaviour change, including future users. Particularly, when BCTs are implemented using AI techniques, the transparency and adherence to the user’s needs become vital, guided by recent regulations (12).

The research presented in this article was conducted as part of developing an AI-based health-promotion intervention application for multiple health behaviours named StarCoach (13, 14). This was done in a participatory design process conducted in collaboration between a research institution and a regional healthcare organisation. During the participatory design process and in previously conducted studies (15–17), a recurrent theme was how to best support behaviour change, which also included integration of the habit to use a mobile application for lifestyle intervention, such as the StarCoach application presented in this article. The adherence to using lifestyle intervention applications is low (10, 11, 18, 19), with an average dropout rate of approximately 50% reported in digital intervention studies. Multi-domain interventions show a slightly higher adherence of 61% on average based on the study by van Kolfschooten and van Oirschot (10), with dropouts occurring mainly during the first phase of studies.

The purpose of the research presented in this article was twofold: (i) to design and develop a multicomponent intervention taking the complexity of multiple domains, suitable BCTs, and MoAs into consideration; and (ii) to study the onboarding phase to uncover factors that may be targeted to improve retention. The onboardíng phase is defined as the first 4 weeks of using the application. A qualitative user study was conducted by engaging eight participants spanning across ages and professional backgrounds.

The main contributions of the research presented in this article are (i) the AI-based health-promotion intervention application for multiple health behaviours named StarCoach; and (ii) results regarding the adherence to and perceived effects of the intervention with a focus on the initial phase of using the app, which we call in this article the *onboarding* phase.

The article is organised as follows. In Section 3, the participatory design process, the theoretical framework, the instrument for knowledge engineering and software development, and the methods applied to evaluate the onboarding phase are presented. The results regarding the StarCoach application are introduced and the motivations for the design choices are discussed in Section 4. The results of the user study are presented in Section 5. Our results are discussed in relation to earlier research in Section 6, and further discussed in Section 7. Conclusions and future work are summarised in Section 8. We begin by introducing the background and research context of our work in the following section.

## 2 Background

The vast majority of health behaviour interventions are aimed at promoting lifestyles that reduce the risk of developing cardiovascular diseases and mental conditions such as exhaustion syndrome. The research presented in this article was conducted in the context of the Västerbotten Intervention Program (VIP) (1, 2). Multiple lifestyle behaviours have been targeted in the program since the 1980s, including smoking, alcohol consumption, nutrition, and physical activity. Individuals within the population of the region are invited for a health check-up in the year they turn 40, 50, and 60 years old, with a health consultation with a trained nurse to identify health conditions and potential health behaviour interventions. Participants are provided with a health profile, visualised as a star with a number of edges representing factors that can be measured and potentially improved. The program has been successful, impacting general rates of cardiovascular events positively (1). Digitalisation of the check-up has recently been completed and a decision-support system has been developed for the nurses.

To complement the development of VIP, the research program Star-C, within which this research was conducted, targets the development and effects of a digital intervention that could support health behaviour change between health check-ups when the person is not receiving healthcare and is consequently not a patient. The unique position of such an intervention as a part of a larger battery of interventions freely accessible by all citizens that are already up and running was seen as an added value when compared to existing health-promotion applications. While an attractive prospect, the challenge is the organisation of the healthcare system, considering that such an intervention would be used primarily by non-patients. Therefore, the approach taken in the Star-C program was to engage a broad multidisciplinary team bringing perspectives of clinicians, citizens, epidemiologists, ethnologists, and health economists, among others.

A participatory design approach was chosen to engage the professionals in design decisions throughout the process. One of the aims of participatory design is to democratise the development of instruments to be used in work environments, by engaging future users and also domain experts in the design process. This approach was also applied in the development and implementation of the regional VIP programme, where nurses are educated in conducting the health check-ups and in using the instruments and are also engaged in developing and modifying the instruments. The development of the Star-C intervention is also integrated into some parts of this continuing VIP development, which is further presented in the Methodology section.

## 3 Methodology

StarCoach was developed through a participatory design process involving a multi-professional team from academia and healthcare, including experts in medicine, psychology, nursing, ethnology, social work, epidemiology, nutrition, and health economics, with researchers in AI, human-computer interaction (HCI), and UX design leading the process. Further, participants representing the user target group were engaged, comprising residents who have or will participate in VIP at ages 40, 50, and/or 60 (20).

In the participatory design process, the framework for developing and evaluating layered interactive adaptive systems presented by Paramythis et al. (21) was adopted by the multidisciplinary participatory design team to outline the modularity of the envisioned knowledge-based person-tailored support system and to frame the topics in design workshops. Following the framework, the *adaptive theory* for the StarCoach application and the related values were defined. The adaptive theory describes how the developed adaptive behaviour of the digital coach will provide the user experience of *control, sense of agency, competence, relatedness, trust*, and sense of *companionship* when using the system and pursuing objectives in desired activities. These aspects form the basis of the set of defined values for the development of StarCoach.

**Figure 1:**
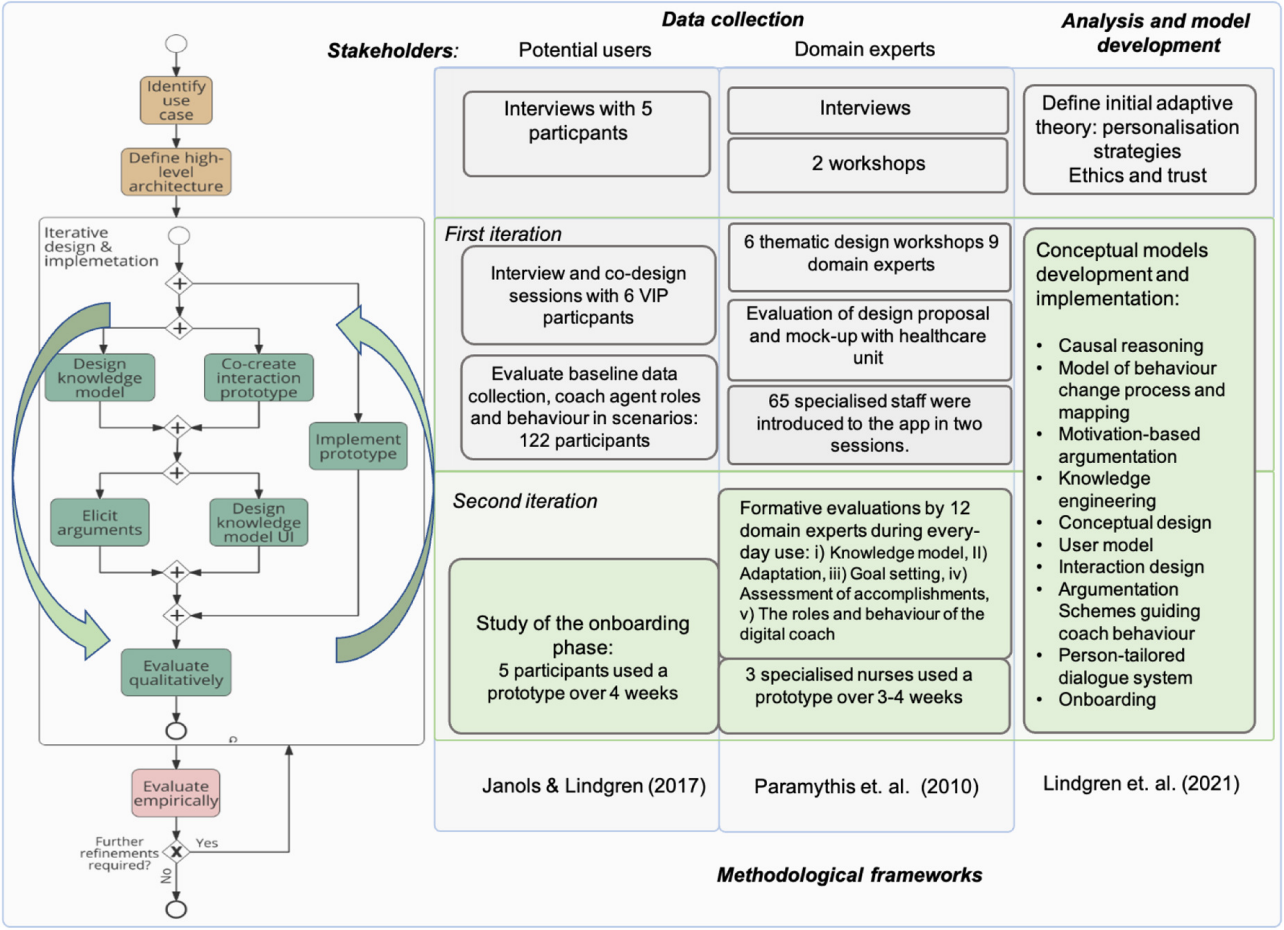
Design process where the second iteration of involving stakeholders is the focus in this article, and the outcomes of the conceptual model development and design materialised in the StarCoach application. Adapted with permission from “Design methodology process diagram” by Helena Lindgren, Timotheus Kampik, Esteban Guerrero Rosero, Madeleine Blusi and Juan Carlos Nieves, licensed under CC BY 4.0.

The participatory design process followed the design methodology for argument-based health information systems presented by Lindgren et al. (22) over three phases, as illustrated in Figure 1. The first phase (upper level in Figure 1) aimed at identifying the major purposes and use cases and defined the high-level architecture. The results are presented in Lindgren et al. (13).

Next, an iterative cycle of design and re-design paired with knowledge elicitation and engineering took place, as illustrated in Figure 1. The first iteration developed conceptual models, mock-ups, and early prototypes, which were evaluated and further developed (15, 16). In the participatory design sessions with target end users in the first iteration, the methodology presented by Janols and Lindgren (23) was adapted, initially, to suit the situation during the COVID-19 pandemic with meetings at distance. The results are presented in Lindgren et al. (17).

In the second iteration, the focus of this article, design choices were converged to implementations of particular functionalities in prototypes evaluated by participating experts and target users. In Section 3.4, the methodology is outlined for the study presented in this article, which focuses on the onboarding phase and is intertwined with the participatory design process. In the following section, the theoretical framework for design and analysis is presented.

### 3.1 Theoretical framework

The intervention design drew on theories of motivation and behaviour change, particularly self-determination theory (24) and the transtheoretical model (TTM) of change (4). A systemic view informed by Lindgren and Weck’s (25) conceptual model guided the factors affecting behaviour change.

The theoretical framework for analysing observations of use and interviews was based on activity theory, in particular, on models of how humans develop skills and internalise the use of new tools (26–28). Identifying the *object*, i.e., focus of activity, as distinguished from *tools mediating* activity, is instrumental. The activity theory zone of proximal development (ZPD) model was applied to assess the participants in relation to the use of the application and activities targeting behaviour change. ZPD is defined as the space in which the activity is too difficult for the practitioner to conduct on their own but can achieve with the guidance of a more knowledgeable peer (29). During observation, the researcher acted as the more knowledgeable peer if the use of some functionality of the application was assessed to be in their ZPD. Situations where the application was not behaving as expected, leading to breakdown situations, were assessed to explore the reasons for and strategies to address such instances. Activity theory also provided a framework to assess the role of the digital companion embedded in the application and how this developed (30).

The combination of these theories provided the foundation to capture and implement support for both everyday decisions and long-term directions to change and maintain a healthy lifestyle, and connect these two perspectives in interaction design and computation to generate tailored support.

### 3.2 Instrument for knowledge engineering

The choice of building the reasoning and decision-making (AI) engine on argumentation frameworks was motivated by the transparency gained by the approach, since this allowed us to represent, identify, and manage arguments motivating and contradicting health choices.

**Figure 2:**
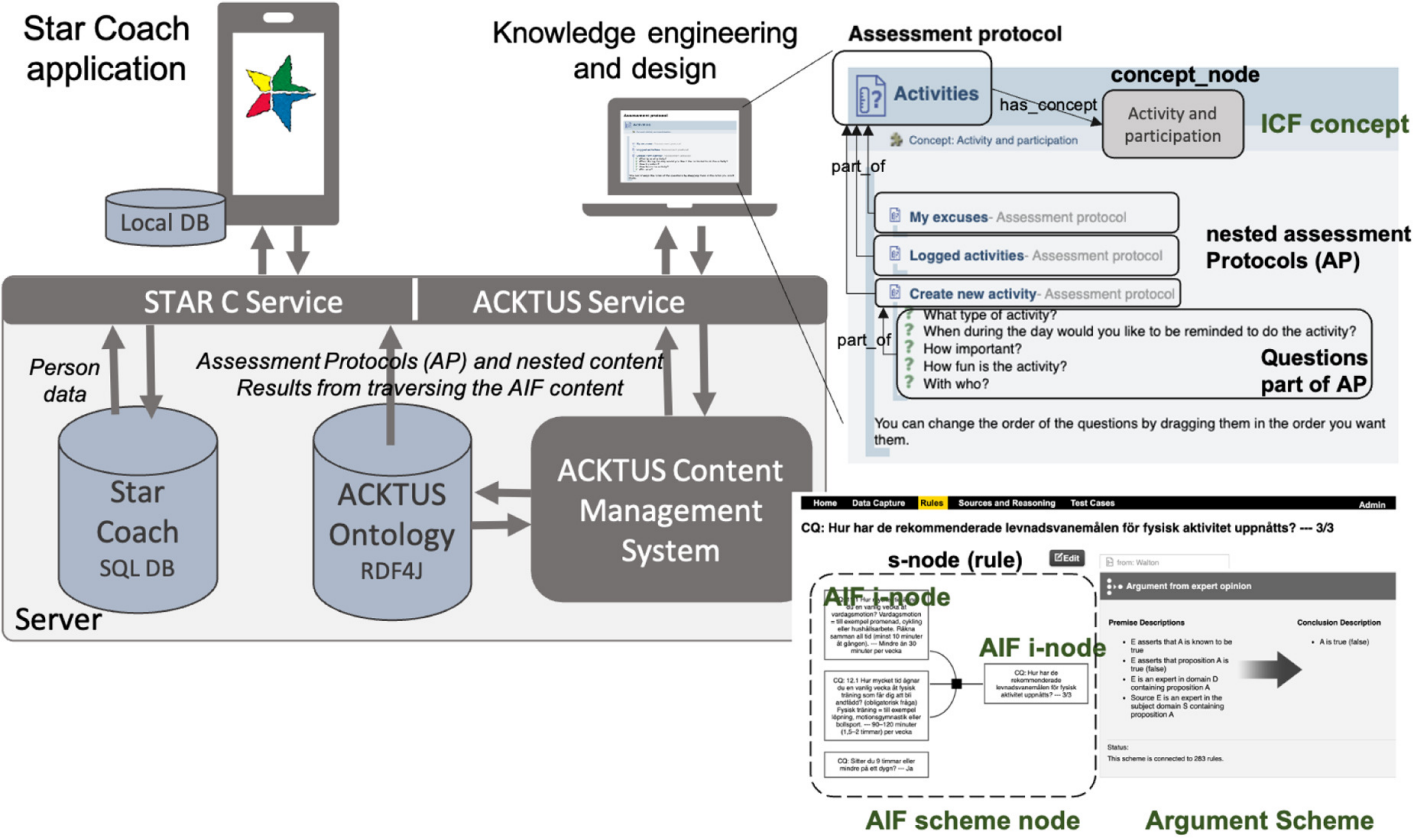
The StarCoach architecture, including the content management system ACKTUS for knowledge engineering and interaction design. Screenshots from: ACKTUS, a system that we built as a part of a research project.

Content management in the application is done using ACKTUS, a Semantic Web application embedding an ontology (a knowledge graph built on OWL^1^/RDF1^2^) that captures medical information, health knowledge, and building blocks of the StarCoach application (31). Reasoning and decision-making are built into the knowledge graph through an adapted version of the Argument Interchange Format (AIF) (32) implemented in ACKTUS. The ontology is stored in an RDF4J repository^3^ and is accessed through an API for content modelling and for generating the content of the application at run time. An example of how an AIF scheme node (s-node) is visualised to the user is shown in Figure 2.

A domain knowledge model specific to StarCoach was defined as an extension of the general knowledge model based on the International Classification of Functioning, Ability and Health (ICF).^4^ ICF concepts are connected to instances of classes, building the content of the application, as exemplified in Figure 2.

The information model of the StarCoach application is organised as a hierarchy of nested or interlinked instances of the class *assessment protocol* (AP) of the ontology. In Figure 2, the AP labelled *Activities* is exemplified, which represents one of the tabs in the StarCoach application. An AP contains an ordered list of content of the types of APs, namely, *questions* connected to a *scale*, or *information*. When the user activates an AP, the content is presented in a step-wise manner, and depending on the answers to the questions, follow-up-APs, questions, and information will be presented. User-specific information, such as answers to questions, is stored in a relational database, along with the ACKTUS AP-ID representing the application-related activity, ACKTUS IDs of questions, and ACKTUS IDs of answer alternatives.

Domain knowledge and assessments generated by the system were evaluated by domain experts in addition to 65 specialised nurses.

### 3.3 Software for mobile application implementation

The StarCoach application was developed using Dart and Java while leveraging the Spring Boot framework for back-end services and Flutter for the frontend. The back-end API was designed following the representational state transfer (REST)^5^ principles and deployed on an in-house server, while the frontend was optimised for the iOS and Android platforms.

For data storage, a MySQL database was used due to scalability and structured queries. For local caching and persistence, an SQLite database was used. The SQLite database is also employed by the embedded coach for activity reminders and quick retrieval of user information. In addition, the application uses a local notification library that schedules notifications for the day once the user logs in.

The software stack was chosen for its scalability, compatibility, and ease of development for mobile devices.

The application was developed for dual-language use, namely, Swedish and English. The participants in the study were Swedish-speaking and used the Swedish version. ACKTUS was used for modelling content in the two languages.

### 3.4 Study design

The participatory design process continued in the second iteration illustrated in Figure 1, with a formative evaluation of prototype versions, which 12 domain experts used over days and/or weeks between meetings. They provided reflections, suggestions, and collectively directed the continued development. A document shared on the web was used for collecting comments during use. Meetings were scheduled on a regular basis, in which comments and experiences were discussed. Changes of content, design, and functionality were made to the prototype between meetings. The main topics were the knowledge model, adaptation, goal setting, assessment of accomplishments, and the role and behaviour of the digital coach. Preliminary results obtained in sessions with participants and VIP nurses with a focus on the onboarding phase were also communicated to participating domain experts in meetings and design choices were made based on this information.

The following research questions were addressed in the study of the onboarding phase:

1. How is engagement in the application developed over the first weeks of use? What are the factors that facilitate and hinder development of engagement?
2. How do participants engage with the app? Are there different ways in which they engage? How does this relate to readiness for change?
3. How are different modules implementing behaviour change techniques used in the onboarding phase?
4. How do participants manage their conflicting goals with the help of the digital coach in the initial phase?
5. How do the participants explain and make sense of the coach application’s reasoning and decision-making related to suggestions the coach delivers?

#### 3.4.1 Participants and recruitment

**Figure 3:**
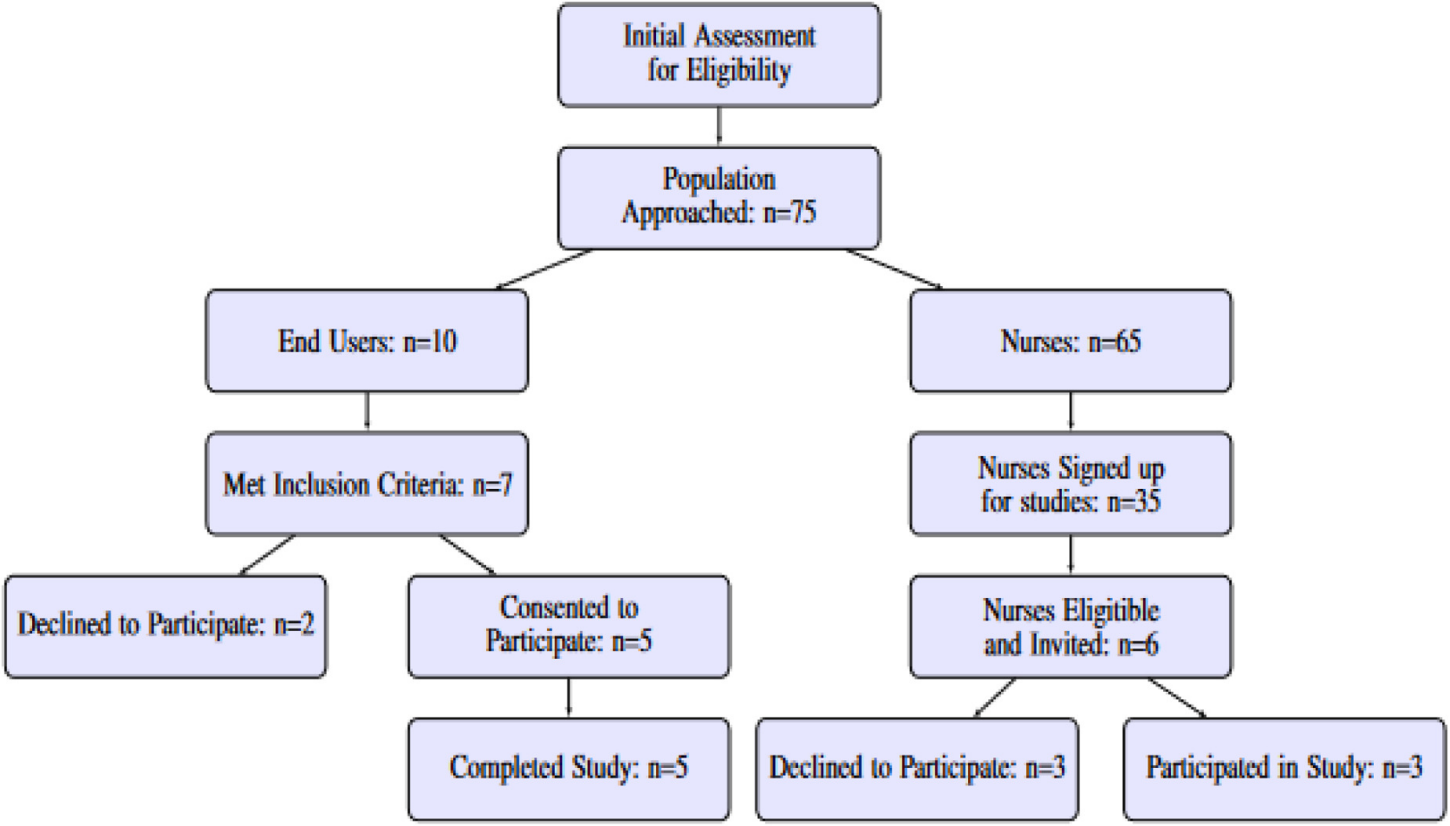
Flowchart of participation selection for the study.

Recruitment of participants was conducted through community-based convenience sampling from participating healthcare clinics and hospitals for the VIP nurses and the Västerbotten region of Northern Sweden for regular participants. The recruitment criteria included residents above the age of 30 as VIP is for people aged 40–60, spoke Swedish, and used an Android smartphone. All the participants provided written informed consent before participation, and ethical approval was obtained through the Swedish Ethical Review Authority (Dnr 2019-02924). A flowchart of the participant selection process for the study can be seen in Figure 3.

VIP nurses were recruited among a group of 65 people who had been introduced to the application at one of two workshops for healthcare professionals. Of these, 35 healthcare professionals signed up to participate in the evaluation studies. As a first step, six of these from selected primary care centres were invited to participate and of these, three confirmed and downloaded the application. The first sessions with the nurses were scheduled 4 days after downloading the app, and the next after 3–5 weeks.

Five regular participants (four female and one male) in the age range of 33–64 volunteered to participate. The participants were unfamiliar with the project, however, three of them had taken part in VIP once or three times, depending on their age, and were familiar with the star profile concept. Three sessions were scheduled: (i) introducing the application and initial observations; (ii) follow-up for use of the application after 4–5 days; and (iii) another follow-up after 3–4 weeks of use. The purpose was to follow the onboarding phase.

The small sample size was deemed adequate for a qualitative exploration study following an onboarding phase deriving from the principle of “information power,” which is an assessment of valid sample sizes in qualitative studies that emphasises well-defined study aims, sample specificity, use of established theory, quality of dialogue, and analysis strategy (33). In addition, a satisfactory qualitative “saturation” was achieved through an adequate number of interviews with the participants, as 9–17 interviews or four to eight focus group discussions are typically deemed enough according to the study by Hennink and Kaiser (34).

#### 3.4.2 Data collection and analysis

A semi-structured interview guide was prepared. The interview guide contained questions on the main functionalities: the star and its role, activities, and the digital coach and its roles and behaviour.

Furthermore, questions regarding the three different ideal types of users outlined in an earlier study (35) and in Section 4.4.3 based on readiness to change were posed to participating nurses. In particular, these focussed on how they viewed the different ideal types, whether they recognised them, and how they thought each would think about using the application.

Each session took approximately 1 h to complete. Sessions with the participants were recorded and transcribed verbatim. Observations of application use were documented through note taking.

Interview and observation data were analysed inductively (36), supported by the theoretical framework. The researchers repeatedly reviewed transcripts, making initial annotations to identify potential patterns. Coding highlighted unexpected or novel themes through an iterative process, following Braun and Clarke’s (37) six phases: data familiarisation, code generation, theme identification, review, definition, and reporting. To reduce bias and strengthen rigour, we maintained an audit trail of analytical decisions and used individual journaling for cross-comparison. Discrepancies were resolved and saturation was determined when no new codes or themes emerged.

Data collected through the use of the application were analysed quantitatively. Patterns emerging in the data were further explored qualitatively to explore potential reasons for their occurrence.

Given the exploratory nature of the onboarding study and limited sample size (*n*=8), we focussed on descriptive statistics to assess user engagement across participants.

## 4 Results: design and implementation of StarCoach

The StarCoach application is aimed to function as a digital coach or companion, embedding the user’s goals and motives into activities, with some level of collaboration with the user. Consequently, general functionalities that enact the values and norms identified in earlier studies can be described and summarised in terms of the following *personalisation strategies* that were identified during the first phase of the design process (Figure 1) (13):

1. Embedded relevant evidence-based knowledge as a base for generating, communicating, and reasoning with personalised information about risks and, potentially fearful, facts: Star Profile module (Section 4.1)
2. Engaging in goal setting to identify an individual’s desire and intention to change behaviour: Activity module (Section 4.2)
3. Avatar as a coach to provide reminders and feedback and to mediate social and emotional support: Digital Coach module (Section 4.3)
4. Assessment of progress in interaction with the user and personalised, interactive visualisation of progress or non-progress, feedback and rewards to increase motivation and support the construction of a positive self-image: Personalisation module (Section 4.4)

These personalisation strategies conform to design principles for behaviour change systems defined in Oinas-Kukkonen and Harjumaa (5) (tailoring, dialogue support, social support, credibility, self-monitoring, simulation of desired goals, or connection between causes and effects). They also cover the values and social norms identified in the design process of StarCoach further explored in Lindgren et al. (17).

The resulting set of activities that emerged from using the digital coach as an actor in the participatory design activities were as follows: prioritise activities, collect data, monitor progress, motivate, encourage, remind, question, challenge, and sustain healthy behaviour and engagement in behaviour change progress.

In day-to-day use, StarCoach supports four general activities: (i) defining goals and related activities; (ii) collecting data and mobilising motivation; (iii) providing overviews of progress, in the long-term perspective or day-to-day perspective; and (iv) providing personalised advice, challenges, and reminders of what motivates the user.

The system supports goal setting and habit formation by supporting the user in defining day-to-day activities. Since the formulation and an ambition level are up to the person to decide, formulating “baby-step” goals is possible, which was seen as highly useful and unique. Goal setting builds on a conceptual model of activity (38). Adherence to the activity goal is supported through a checklist of what the user has aimed to do, in which they also log activities, and through reminders delivered by the digital coach.

The system provides assessments of activity progress and accomplishments and encouraging messages. Patterns over time are shown in a graphical format.

The system adapts dialogues to the person by ordering the goals within a view and selecting the most suitable message based on the situation. The message could be a cheerful positive message of encouragement, contain an activity argument with a reminder about the person’s motives for the activity, or contain an activity argument based on medical or health domain knowledge. The initial formalisation and implementation of the agent’s behaviour and actions are presented in Kilic and Lindgren (15).

The functionalities that emerged from the participatory design process corresponded to the following major areas of BCTs (8): Goals and planning (1), Feedback and monitoring (2), Social support (3), Shaping knowledge (4), Natural consequences (5), Comparison of behaviour (6), Association (7), Behaviour regulation (8), Comparison of outcome (9), Reward (10), Regulation (11), and Self-belief (15). A mapping was done of the embedded functionalities to BCTs to identify which ones the participants used (Table 1).

**Table 1:** An overview of the behaviour change techniques embedded in the three StarCoach modules.

Behaviour change technique	Star profile	Activity module	Digital companion
1.1. Goal setting (behaviour)			
1.2. Problem solving			
1.3. Goal setting (outcome)			
1.4. Action planning			
1.5. Review behaviour goal(s)			
1.6. Discrepancy between current behaviour and goal			
1.9. Commitment			
2.2. Feedback on behaviour			
2.3. Self-monitoring of behaviour			
2.4. Self-monitoring of outcomes of behaviour			
2.7. Feedback on outcome(s) of behaviour			
3.3. Social support (emotional)			
4.1. Instruction on how to perform behaviour			
4.2 Information about antecedents			
5.1. Information about health consequences			
5.3. Information about social and environmental consequences			
5.4. Monitoring of emotional consequences			
5.5. Anticipated regret			
5.6. Information about emotional consequences			
6.3. Information about others’ approval			
7.1. Prompts/cues			
8.2. Behaviour substitution			
8.3. Habit formation			
8.4. Habit reversal			
8.7. Graded tasks			
9.1. Credible source			
9.2. Pros and cons			
9.3. Comparative imagining of future outcomes			
10.10. Reward (outcome)			
11.2. Reduce negative emotions			
13.2. Framing/reframing			
13.3. Incompatible beliefs			
15.1. Verbal persuasion about capability			
15.3. Focus on past success			

The behaviour change techniques and their number coding refer to those used in the theory and techniques tool: https://theoryandtechniquetool.humanbehaviourchange.org/tool.

In the following subsections, each of the four personalisation strategies is presented along with the formative evaluation outcome of the participatory design from the 12 domain experts. Their comments mainly concerned the following seven themes, which will be elaborated in more detail in the following sections:

• Knowledge model and its visualisation;
• Adaptation;
• Goal setting;
• Assessment and communication of accomplishments;
• Roles and behaviour of the digital coach;
• Ethics and values; and
• Onboarding.

### 4.1 The star profile: a holistic representation and communication of risk, behaviour, and health

A representation of the user’s health status is provided as a general and holistic overview of important areas that contribute to a person’s health. This status is visualised as a star, similar to the star provided to the person by the regional healthcare provider at the health check-up. A snapshot of the person’s health status for each of the five lifestyle domains, namely, *physical activity*; *balance in life* including sleep, stress, and mental health; *nutrition*; *alcohol* intake; and *tobacco and nicotine* use, is provided through the shape of the star’s edges. The star’s edges represent *value directions*, or motives, which can be seen as high-level goals, but represent that behaviour change is a continuing process rather than finished when a goal is reached.

The more healthy behaviours a person reports in the domain represented by the edge, the more filled the star edge is. The levels of accomplishment are also colour-coded, ranging from dark green for healthy behaviour to orange, representing behaviour that needs to change to improve health. The star represents and communicates a long-term goal to fill the star edges. Definitions of accomplishment are provided by the expert team at the healthcare organisation, guided by evidence-based knowledge and national guidelines and modified and updated since the beginning of VIP. As such, the star is normative, and serves as an extrinsic motivator, reinforcing the norms identified in an earlier study (17).

**Figure 4:**
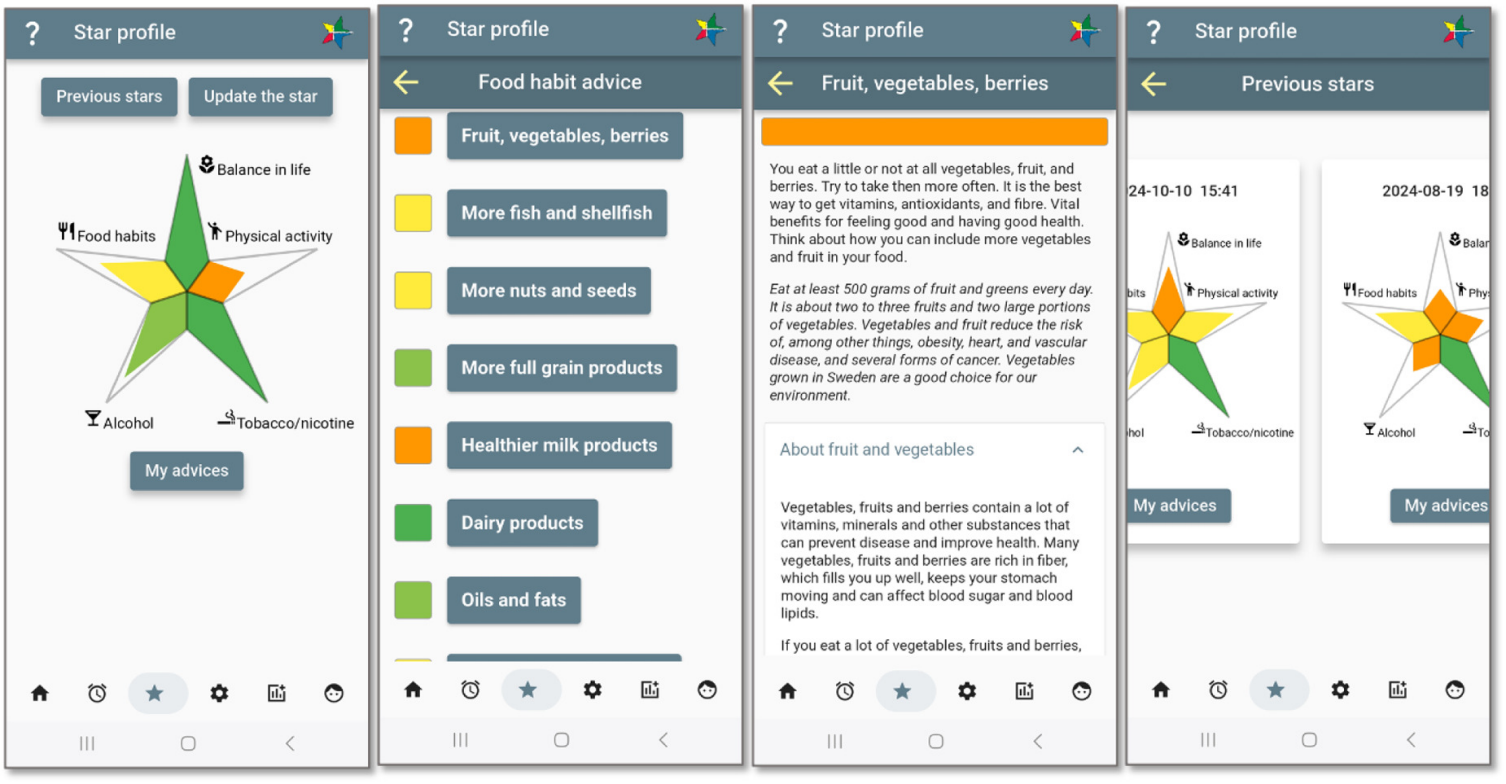
A holistic view on the star-shaped health status. Screenshots from: StarCoach application.

Assessment of the health status is based on a set of questions that the user answers when initiating the use of the application. The answers are analysed based on algorithms developed by the regional healthcare provider and based on national guidelines and medical knowledge. Four of the experts in the design process were also responsible for developing these algorithms and tailored advice based on the answers, which are also provided in StarCoach (e.g., Figure 4). The user can update the star anytime by providing new answers to the questions on which the star is built. The user can also view generated stars in the past to compare and assess progress over time.

#### 4.1.1 Reasoning about and reflecting on evidence-based knowledge, risk assessment, and recommendations

The health dialogue, embedding the motivational interviews that the nurses have with each client in VIP, covers the person’s health status and its consequences and provides guidance on how the person may want to address their health issues (39). The evidence-based and best-practice knowledge utilised in VIP has been crafted by the team of medical and health experts since the inception of VIP in the 1980s in dialogue with the community of clinical staff engaged in health check-ups (1), and has also been revised over the years as knowledge and national recommendations have developed. The knowledge was translated into a decision-support instrument for nurses that was initially paper-based, and which has recently been digitalised as part of the digitalisation of data collection during the health check-up.

StarCoach embeds relevant evidence-based and best-practice knowledge as a base for generating, communicating, and explaining personalised information about risks and potentially fearful facts. This content is a subset of VIP decision support adapted to be useful to individuals outside of healthcare. The knowledge was also revised and updated during the knowledge elicitation and knowledge engineering phases during the implementation of StarCoach, as VIP content was updated when medical knowledge and national guidelines on healthy lifestyle habits evolved. Consequently, a large proportion of comments in the participatory design process related to the knowledge content and its revisions and how to communicate the knowledge to the user.

For each lifestyle domain except balance in life, person-tailored recommendations are generated based on VIP and colour-coded in the same scale as the star. For nutrition, the system follows the national guidelines for each of the 10 sub-domains to which the person may comply differently, and thus different colour-coded assessments are also provided (Figure 4).

**Figure 5:**
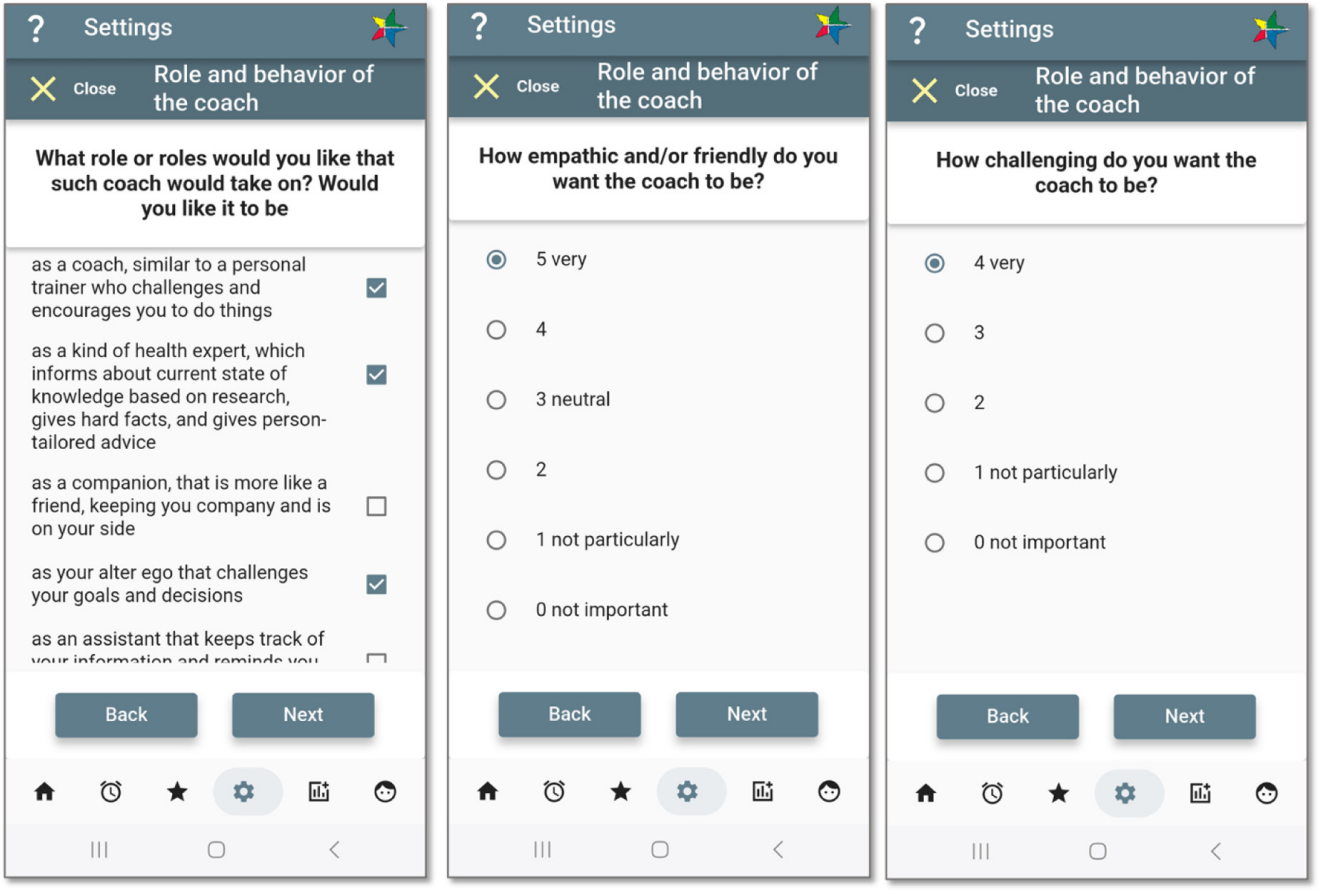
Shaping the coach. Screenshots from: StarCoach application.

The generated, person-tailored advice communicates the level of risk and benefits of changing behaviour. For each piece of advice, generic information and suggestions are provided with links to external sources. In addition to the advice view connecting to the star, communication and reflection on medical knowledge underpinning the advice are also done in dialogues with the digital companion. When the user selects the role of expert for their digital companion, or mixed role as a coach (Figure 5), the messages from the digital companion partly connect to the content of these pieces of advice.

Aspects relating to the “balance in life” domain are communicated in dialogues between nurse and patient in VIP, but are not yet embedded in the decision-support material, and thus also do not generate particular advice in StarCoach. This was commented on by both participating nurses and others in our study, who also wanted advice on balance in life matters. As a consequence, advice was also developed for this domain, but initially, this was formulated as generic advice.

### 4.2 Activities: setting baby-step goals and monitoring achievements

**Figure 6:**
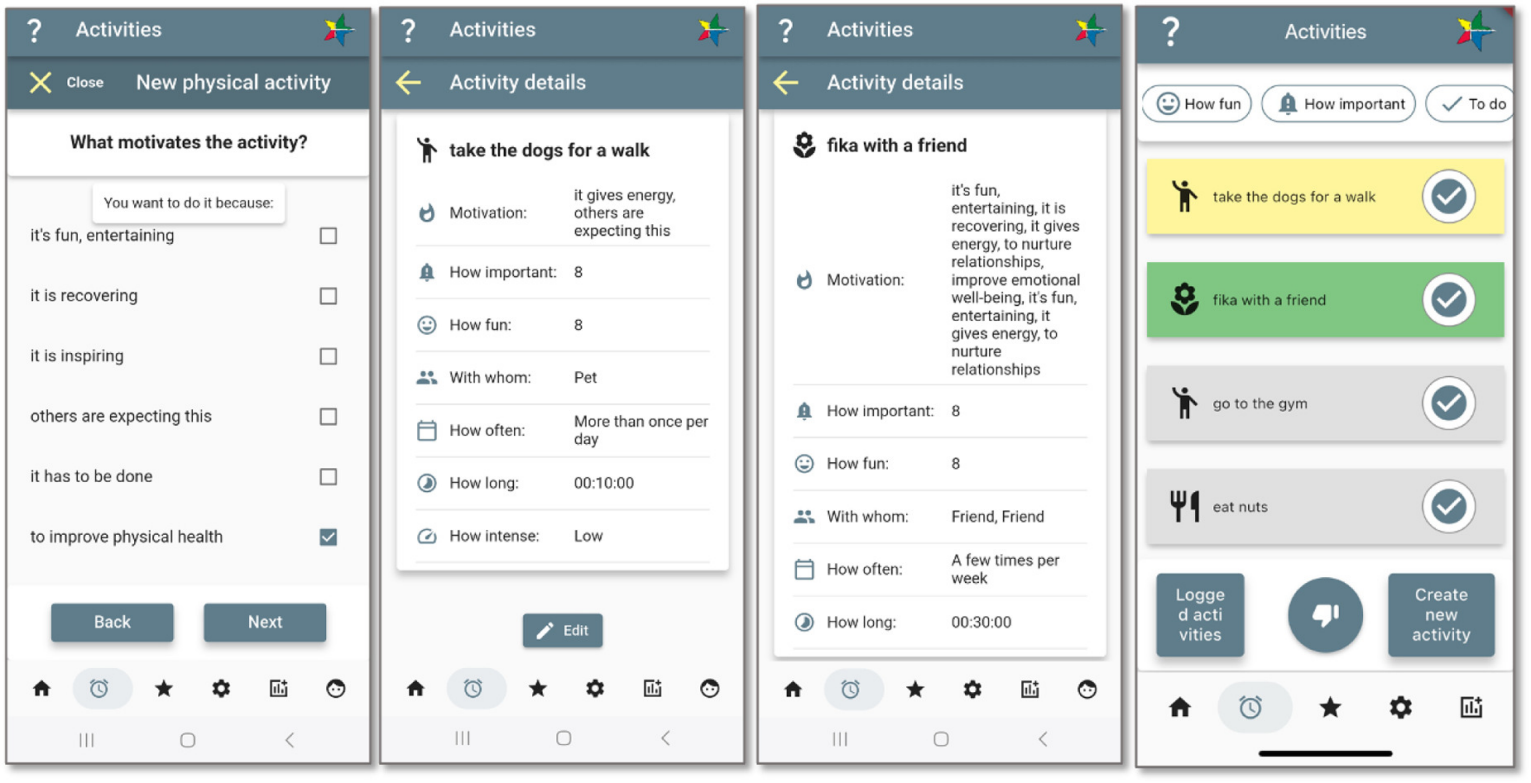
Activity definition and goal setting, corresponding to domains in the star profile. Screenshots from: StarCoach application.

Study participants in an earlier study described successful behaviour change taking place when there was a structure and a social context in which the behaviour change took place, e.g., a child telling a parent that smoking is bad for them or someone losing weight as a teamwork effort with a partner who needed to lose weight before a medical intervention (40). Such reasons are captured by initial questions posed by the application about what motivates the desire to change behaviour. Furthermore, support to set small and relevant sub-goals was seen as important. Consequently, providing support to connect motives to related sub-goals is seen as important in the design of the goal setting. An example of different levels of activity and related goals is shown in Figure 6, where the two exemplified “baby-step” goals have motives related to the value directions of physical activity and balance in life represented in the star profile.

Setting goals is done in two ways. The user initially defines “value directions” as part of the baseline assessment by selecting behaviour(s) targeted for intervention as a high-level goal setting, translated into the star profile. The baseline assessment captures both motives and barriers for change. At run time, the user defines which activities to aim for when targeting their value directions (where also the inclusion of others in their social environment is elicited) and sub-goals, referred to as “baby-step goals,” relating to the value directions.

**Figure 7:**
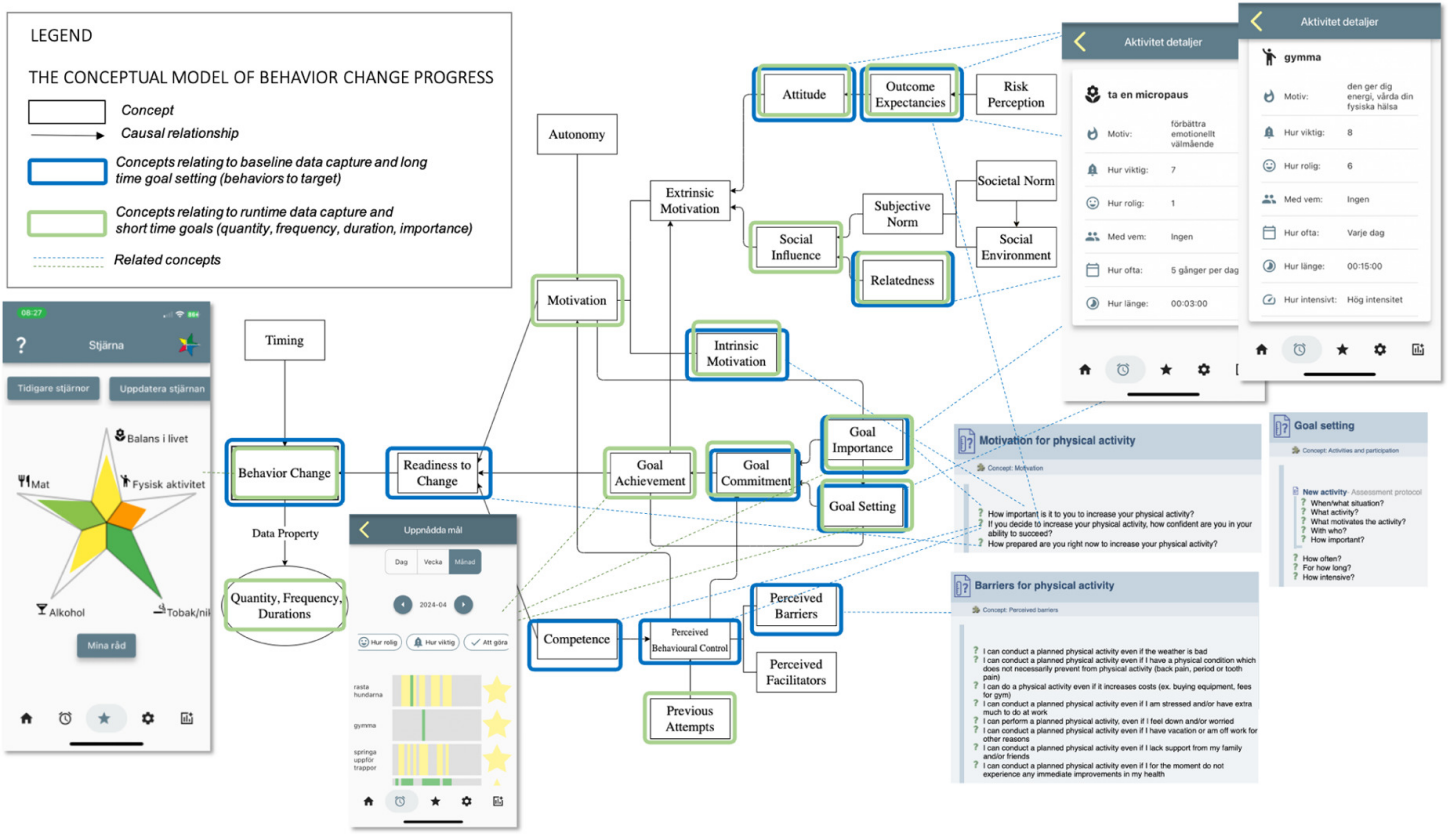
StarCoach information mapped to the conceptual model of behaviour change progress. Adapted with permission from “Jonglera information mapped to the conceptual model of behavior change progress” by Helena Lindgren and Saskia Weck, licensed under CC BY-NC 4.0. Screenshots from: StarCoach application.

Specific activities, aimed to be conducted on a regular basis for habit formation, are assigned the quantitative measures *frequency*, *duration*, *importance*, and *how fun*, which can serve as “baby-step” goals, and qualitative information, such as underlying motive(s) and whether someone else is involved, to capture whether the activity is also a social activity (*Social Influence*, *Relatedness* in Figure 7). The information is used by the coach module to build arguments as motivating statements in dialogue with the user to promote goal commitment, to which the user may respond with a thumbs up as confirmation/agreement or a thumbs down for rejection/disagreement.

Activities are listed as a checklist of to-dos and are colour-coded where grey means not initiated, yellow means activity is in progress, and green represents the activity is accomplished according to the intentions of the user. The user can monitor their progress through overviews of the colour-coded activities they have logged in a day, week, and month view. They can also sort activities based on lifestyle domain (default), level of fun, importance, and accomplishment, to self-assess reasons for progress. For instance, a person may prioritise and conduct fun activities before activities with higher importance or those for the targeted lifestyle.

When the user logs an activity, a positive response is given along with follow-up questions tailored to the targeted lifestyle domain, which can be answered if the user wishes. Depending on how engaged they are, they may want to, along with the quantitative measure of accomplishment, also evaluate the quality of accomplishment, e.g., how they feel after a recovery activity or physical activity. For alcohol, tobacco, and nicotine, if desired, they can log consumption for monitoring purposes.

### 4.3 The digital coach: role, position, and behaviour

In earlier studies, potential users expressed mixed expectations of the digital coach’s behaviour. On the one hand, the coach had to be mature and serious but also entertaining and encouraging; it should not command change. On the other hand, it has to be commanding to have some impact (16, 40). Some participants indicated that they wanted to be sympathetic with and like the coach to adhere to advice. One participant even expressed that being able to choose between different versions of the coach would help. A conclusion from the study was that personalisation is very important in the development of adherence in the usage of the application.

Based on previous studies, functionality was included that allows the user to shape the role(s), character, and behaviour of the coach in StarCoach (Figure 6) to increase motivation and control of the system. In addition, the user can also select their preferred coach role among the following options: coach, expert, companion, alter ego, and assistant. The coach’s character or behaviour is defined by answering two questions about how empathetic and how challenging it should be. The implementation and evaluation of this module is presented in Kilic and Lindgren (15).

The coach is implemented using a rule-based reasoning system using OWL/RDF ontology and AIF-based dialogue structures (Section 3.2) [technical details can be found in Kilic and Lindgren (15)]. The choice to opt for a rule-based approach rather than generative was due to traceable and transparent decision-making in alignment with ethical AI guidelines. Furthermore, it was agreed with domain experts that a rule-based approach was superior in this case to mitigate the risk of dubious healthcare advice.

**Figure 8:**
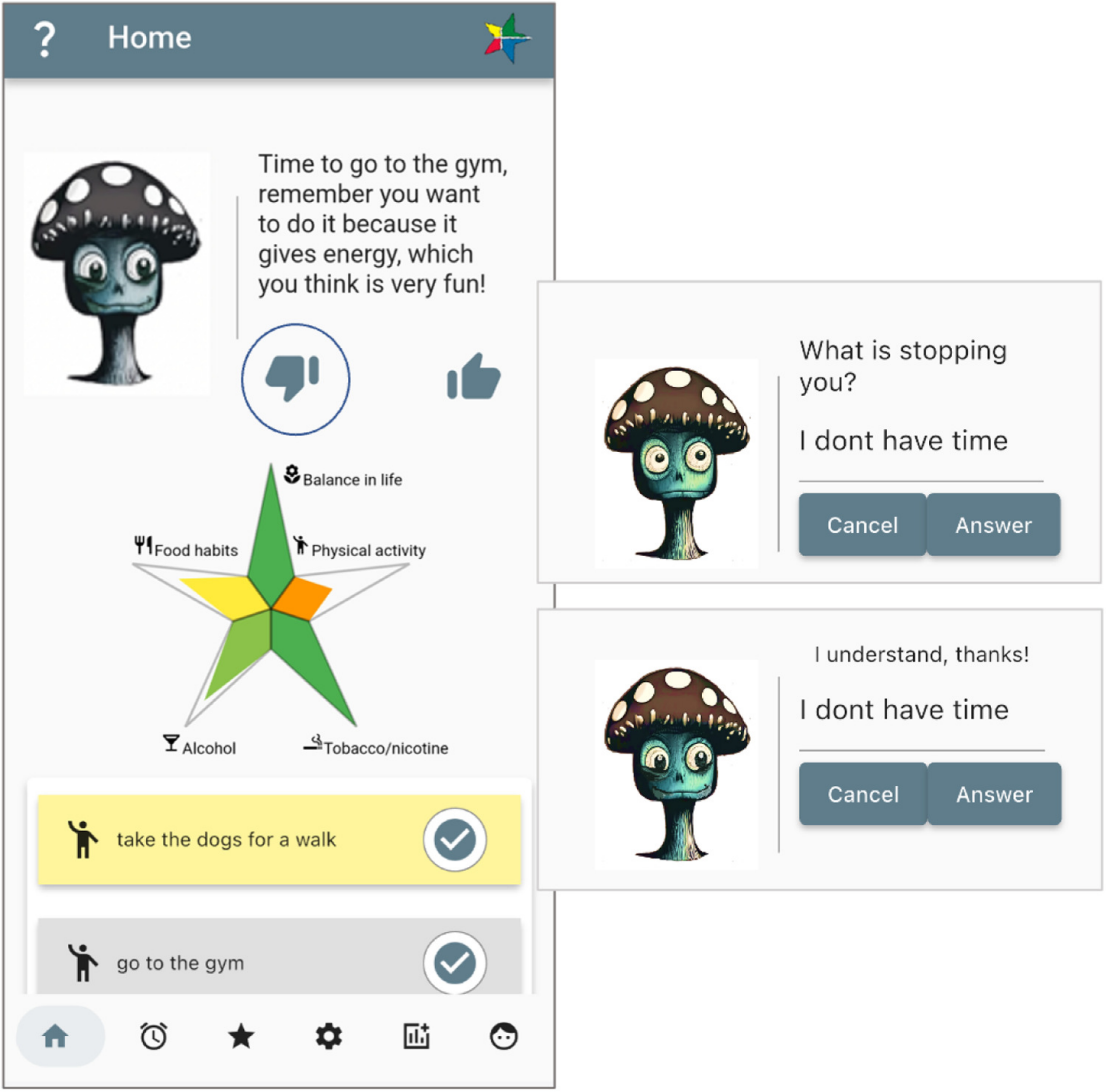
Micro-dialogue with the coach. Screenshots from: StarCoach application.

In terms of embodiment, the coach is visualised as an animal (cat or dog) or a mushroom (Figure 8). Digital agent embodiment plays a large role in adherence to usage and interaction, and different users have varying preferences in coach types, which have shown effects (41, 42). Therefore, having an agent that can be interacted with, is responsive, and can be chosen by the user is an important aspect of the application. The coach figures in the StarCoach application respond to user input non-verbally through expressions, along with dialogue lines fitting the situation. For instance, when interacting with the coach in dialogue, it changes to a happy expression when the user responds positively and to a slightly worried or sad expression when the user responds negatively. Furthermore, addressing comments from participants, the coach can also be put to sleep.

The position the coach takes depends on the preference of the user for that particular topic and its activity type and can either be a supportive position or a challenging position. An example, for clarity, is the physical activity “running.” The user’s preferences for “running” are based on the user’s goals for the physical activity which, in this example, will be “very important” and the user’s readiness and desire for change in the physical activity and the situation. The coach, equipped with past dialogue lines between itself and the user, amalgamates this information and chooses a supportive or challenging position. For the coach to take on a challenging position, the user must have chosen at least some challenging value in the question “How challenging do you want your coach to be?” (16).

### 4.4 Personalisation module

Operational and physical constraints were identified in the sessions with the multidisciplinary team of experts using the model for layered adaptive intelligent systems (21). The relevant layers are the collection of input data, interpretation of data using static and dynamic models, deciding upon adaptation and implementation of adaptation decisions, and design of user interfaces mediating the collaborative activities and personalisation strategies. The following sections present StarCoach from the perspective of these layers.

#### 4.4.1 Collection of data in StarCoach

From an ethical perspective, there is a trade-off between what is the minimum sufficient information for the system to be of value to the user vs. what information to include that is of value from a research perspective. To clarify the two different purposes for the user, a dedicated page in the application contains information about their participation in a study and indicates that they can withdraw from the study at any time. They also received information about the project and study, and a timeline with data collection points and links to questionnaires for follow-up in months 1, 3, and 6 if taking part in the randomised controlled study.

Domain-specific questions deemed necessary for evaluating effects were included at baseline and follow-up in months 1, 3, and 6, partly based on the health check-up content and the related decision support.

The set of questions answered at the baseline assessment captured information relating to concepts in the conceptual model of behaviour change (Figure 7) (25). These covered the prioritisation of which behaviour or lifestyle areas a person would like to maintain or change to secure good health (high-level *Goal Setting* in Figure 7), how important this is (*Goal Importance*), how prepared the person is to change (*Readiness to Change*), and how confident the person is in accomplishing a change if deciding to change (*Competence*, *Perceived Behaviour Control*, *Goal Commitment*, *Outcome Expectancies*). When selecting prioritised health behaviour(s) to change, additional structured questions relating to self-efficacy are posed, including perceived potential barriers relating to the specific chosen health behaviour (e.g., weather or pain conditions in the case of physical activity); financial, psychological, contextual/situational barriers; and if there are no immediate health rewards (*Perceived Barriers*). Arguments for and against a particular health behaviour are also specified, either by selecting among the predefined arguments or formulating one’s own (*Attitude*, *Outcome Expectancies*). Both extrinsic and intrinsic motivators are identified (24).

Data are collected during everyday use when the user conducts the following tasks (formalised as an assessment protocol in the ontology): create or update an activity, answer follow-up questions after conducting an activity, update their star profile, change preferences regarding the coach, and explore advice generated relating to information contained in the star.

When creating an activity, one or more motives are captured, which represent extrinsic and intrinsic motivators (24). To capture the social character and value of the activity, the user also notes whether the activity will typically be done alone or with someone.

Further, a log of activities and dialogues with the coach is stored, including reasons for why the activity was not conducted.

In addition, some click events are logged, which indicate exploration of information embedded in the app.

#### 4.4.2 Interpretation of collected data

An initial interpretation of the data at baseline is done by the system to assess the level of accomplishment in relation to value direction and the five domains of behaviour, which is visualised in the star profile. Related person-tailored advice is also generated and accessible by the user when opening the application for the first time. As mentioned, this is done by the system based on the AIF (32), which is implemented in the ontology underpinning the application.

During run time, the user can choose to update the star profile, which will also update the set of advice. The user’s log of activities is computed to generate accomplishment levels relating to each activity, following the user’s definition of accomplishment (number and frequency of occasions).

Preferences regarding what role the agent should have guide the generation of the coach’s behaviour. Preferences regarding the level of empathy and challenging behaviour are used to generate a role/character or alternate between characters based on the user’s preferences. Behaviour and roles are related to argument schemes and implemented based on AIF (32, 43).

Domain knowledge and assessments generated by the system were evaluated by domain experts in addition to 65 specialised nurses. The knowledge model and interpretations of the data were updated accordingly.

#### 4.4.3 Deciding upon adaptation and applying decisions on adaptation

Decisions on adaptation were defined by domain experts based on domain knowledge and modelled in ACKTUS as instances of scheme-nodes in the adapted AIF ontology. Decisions (e.g., person-tailored advice) are then generated by the system based on these scheme-nodes consisting of premise nodes and a conclusion node, and are sometimes assigned a strength value.

Further, the personalisation module adapts the companion’s behaviour and actions to the user’s profile and preferences.

Three levels of readiness to change were identified and approached by nurses differently in the motivational interviews. These are summarised in italics in the following list. We describe for each level how StarCoach functionality can map to the approaches applied in the motivational dialogues:

1. *Low readiness to change: offer information by elaborating on drawbacks and carefully explore the advantages of change*. This is initially done in StarCoach at baseline by asking the user to outline the arguments in favour of the healthy behaviour and factors that prevent them from adopting the desired healthy behaviour. A selected set of advantages and drawbacks is presented as argument alternatives in questions to guide the user. This is aimed at triggering and support reflection in the user.
2. *Medium readiness to change: develop ambivalence by exploring barriers, difficulties, and solutions, and reinforce change*. In StarCoach, this is continuous throughout daily use as the coach reminds the user about activities and opens dialogues with them to elicit reasons for, and barriers to, conducting planned activities.
3. *High readiness to change: support action through the formulation of clear goals and sub-goals, including plans for making the change*. StarCoach supports this through goal setting and activity creation.

It was discussed whether the application would assess at which readiness level a user is, and tailor the support differently. In the current version, this is not done. Instead, the aim is to embed the three elements and make these accessible to all users.

From the onboarding perspective, it is expected that most participants will conduct the baseline assessment (1), and, if they show low readiness to change, they may not continue using the application to do tasks relating to (2) and (3). In the study presented in this paper, the participants entered at level (2) and were consequently assessed as manifesting medium (2) or high readiness to change (3). How the use of the application reflected their readiness to change will be further explored in future studies.

Based on the discussions in workshops with the specialised nurses, it was concluded that the current version of the system primarily supports the first two levels of readiness to change, prompting the user to take action, rather than boosting already active individuals. In particular, the application does not support setting time points for activities during a day, which some of the nurses perceived as desirable for organising activities.

To summarise, the participatory design process generated results regarding the following concerning the system’s adaptive behaviour:

• How to respond to the user in different situations/activities.
• What to present to the user: select a subset of information.
• How to present data to the user: alternative ways to visualise progress and information generated based on collected data.
• Selecting nudges, reminders, and encouragements, and deciding upon the next move in a dialogue with the user.
• Proactive behaviour: why, when, and how.

The resulting implementation contains the following: visualisation of progress relating to both short-term and long-term goals; statements by the companion regarding short-term goals; star shape to represent accomplishment in different lifestyle domains; calendar block with a coloured scheme for accomplishments related to short-term goals, which also displays colour-coded stars to emphasise success; a digital agent appearing as an avatar representing the agency of the app; activities prioritised by the user are also prioritised by the agent; and positive feedback regarding accomplishments. The request from the companion for a response to messages and feedback is done using two icons: thumbs up and thumbs down. The request for response when an activity is logged is posted as a follow-up question specific to the lifestyle domain to which the activity is associated.

Adaptation of the digital companion’s behaviour is done as follows (example in Figure 8):

• Select visual appearance based on user’s choices.
• Select role and position based on the user’s preferences.
• Based on level of accomplishment, role, and position, select topic for a dialogue (which activity, and purpose, e.g., to remind, give positive feedback, etc.).
• Adapt behaviour (happy, less happy, concerned) depending on the situation.
• Ask for response, and when a negative response is provided, ask for reasons (counter-arguments).
• Selecting a time point to act by is done based on whether the user has responded negatively to a proposed activity, and then this topic is put on hold for a time span of 3–4 h.
• If user does not respond, show “waiting behaviour.”

#### 4.4.4 Assessment of progress in collaboration and interaction design

Satisfaction with the situation, achievements, and activities is assessed to follow up on goals and sub-goals during the use of the application. For each behaviour, a preliminary set of ecological momentary assessment (EMA) questions was defined and modelled in ACKTUS (31). The run-time assessment captures activity-related information specific to the selected health behaviour(s) through subjective assessment of the performed activities.

Long-term goals are visualised in the shape of a star (see Figure 4, Section 4.1).

Statements in the form of advice/recommendations, based on evidence-based medical knowledge and an individual’s information, are provided in short formalised micro-dialogues with the user to increase knowledge and motivation, which the user can also respond to positively or negatively, providing counter-arguments (see Figure 8, Section 4.3).

## 5 Results on the evaluation of the onboarding phase

Five participants (P1–P5) were followed during the onboarding process over 4 weeks, during which they familiarised themselves with the application and explored and internalised the use of the different functionalities. They accessed a version without the baseline or follow-up questionnaires. Further, three specialised nurses (N1–N3) involved in VIP downloaded and used the application over 3–4 weeks.

All the participants used smartphone applications regularly and a few participants were currently using an application for tracking health-related information (physical activity and food intake). P1–P5 were observed when they installed and started to use the application, while N1–N3 did this on their own and they were followed up with at a meeting within a few days. A general observation was the way in which their use of the application mirrored their age, in aspects such as speed, ways they navigated across activities, and finding information in the application. In particular, the participants below the age of 40 were more “fluent” and spent less time thinking about and looking for information.

The five participants (non-nurses) expressed different positions regarding application use in general. While one enjoyed tracking data and filling out questionnaires (P3), another explicitly did not (P4). One did not actually enjoy tasks related to technology, such as downloading and installing apps (P1), while another enjoyed trying new technology (P2).

P1 and P2 showed a high level of readiness to change based on the three levels defined in Section 4.4.3, as they showed less interest in dwelling on reasons for change. Thus, they were already aware of why and what they needed to do, but had not decided to change yet. The application confirmed what they already knew and had to some extent internalised as important. P3, P4, and P5 showed medium readiness to change, since they were aware of the challenges and understood the benefits of, and barriers to, change. In some domains in which change would be needed based on the star, they were not interested in change.

Observations of behaviour relating to unclear layout, information, navigation, etc., informed the development of the app, leading to adjustments in the design of the user interface and information content. In the following subsections, findings are presented relating to internalising the use of the StarCoach application and specified activities relating to lifestyle changes.

### 5.1 Connecting to current activities and routines

P1–P5 had a few physical activities they already performed on a regular basis to maintain or improve their health. Among the first activities they created were those that they already had the habit of doing. Two of the participants (P2, P3) quickly created activities relating to nutrition, which they were already aware needed to change. A third participant (P4) mentioned that she should probably create a particular nutrition task she was aware of, which was orange in the nutrition advice in the star, but did not want to, and deliberately maintained her counter-arguments for why she did not make a change.

Most of the participants also created activities relating to the balance in life part of the star profile, typically recovery activities, which, for two participants, included spending more time with friends.

Only one of the participants (a nurse) created an activity related to alcohol. One of the regular participants wanted to have her star edge for alcohol dark green, i.e., maximally fulfilled, but could not since she was not willing to reduce her intake of alcohol further, which was perceived as frustrating. When informing the participants that if they created an activity that could help reduce alcohol intake, one can also log the number of standard glasses to keep track of consumption, four of five participants expressed a wish to try this. However, during the evaluation period, none of them did.

After 2 weeks, the five participants had a mix of activities with routines and one or two additional activities. By creating these activities, they seemed to form a commitment to conduct them, which was observed when two participants for different reasons did not use the application for a few days, but continued to do the activities despite not logging them in the app: “…One thinks about the activities, since one knows that they are put there in the app even if not using the app” (P2).

After 3–4 weeks, the five participants perceived that the application had contributed to changing their behaviour. P2, P3, and P4 had conducted both their old activities as before and new activities as planned. This was also manifested in their updated star and connected advice, as two participants had one of their star edges turning from yellow to light green, and one participant had one of their nutrition goals shift a level. P1 and P5 noticed a difference in that they conducted their old physical activities more frequently, but this was not visible in the star.

Two of the participants had created a routine to log their activities every day at a particular time (P1 and P3).

Two participants reflected on how conducting multiple one-time activities allowed them to meet their long-term goal to increase physical activity. Currently, the application is not designed to target one-time activities but instead focuses on repeated activities to form new habits.

### 5.2 The meaning of the star

The star was perceived as a very, if not the most, important module in the application by all the participants. The nurses and three of the five participants viewed the star as the ultimate goal image, which could be reinforced more strongly in the application. This could be done by encouraging the user to update the star at least once a month.

One participant expressed that “*The star is representing ‘me,’ I want to be a good person*” (P5), and “*The star becomes my goal*” (P1).

Another participant (P2) was very disappointed with the star he received when participating in VIP 8 years earlier and wanted to improve it. He viewed this application as a chance to do this.

P4, N2, and N3 pointed out that the balance in life domain could be improved, as advice could be added, and the evaluation of the accomplishment level could be improved since this could change between days. This informed the design work by the participatory design team and led to further improvements of the system’s knowledge base.

### 5.3 The digital coach

Notifications were positively received, and it was mentioned that it was pleasant to be greeted in the morning with a “good morning.” A few participants wished for more frequent notifications to help draw their attention to the app.

Being able to select an avatar and the characteristics of the coach and the coach itself were received positively. One of the nurse participants was more negative (N1) and focussed on the visual appearance of the coach. She indicated that she would like to have an option without a character, rather than a thing, to avoid a sense of “childishness.” She was also the only participant who did not add preferences regarding the coach in the end. The role or roles that were selected differed among the seven remaining participants. While one of the regular participants had a neutral view of the agent (P2), the other four had four different perspectives and expectations. One viewed the coach after 4 weeks as a kind of “friend,” or a companion that would pop up and cheer when she did something good (P5). She suggested that one should be allowed to name the coach and have a nickname for it.

Another participant also initially wanted it to be friendly and had assigned all roles with a high emphasis on being empathetic (P3). However, after 4 weeks, she preferred it to be more challenging and hold her accountable to stick to her plans.

The participants responded to the coach’s statements, including providing reasons for why they did not adhere to the coach’s suggestions in the particular moment when a suggestion was received.

### 5.4 Use patterns based on collected data

For participants P1–P5, there was a pattern of continuous usage over time with short periods of non-use in between. P5 had a large gap between February and March due to personal reasons. An observation was that P1 logged activities more frequently than all the others.

The nurses used the application less, and this could have been due to testing the application for patients rather than personal use. N1 and N2 used the application for almost a week before ceasing usage, whereas N3 continued testing for a longer period of time.

There were some days on which every user’s usage peaked, and this was likely due to interviews that took place between the researchers and users on those days in which users tested more functionalities with the researchers. Interestingly, the users preferred using the application more during the weekdays rather than weekends.

During the 4 weeks, the regular participants created 4.8 activities on average and the nurses 3.7 activities. All the participants created at least one physical activity (a total of 18), six of the participants created at least one recovery activity (a total of 8), three of the regular participants and two of the nurses created activities related to nutrition (a total of 6), and one nurse created a tobacco and alcohol-related activity each.

Overall, the regular participants logged an activity an average of 16 times and the nurses 3.6 times during the 4-week period.

In total, 60% of the activities logged by the participants were followed up with questions (nutrition activities did not result in follow-up questions). On average, they answered the follow-up questions eight times per person for physical activities and eight times for those who had created recovery activities.

The participants created new stars 29 times, with 3.6 per person on average.

The participants changed or revisited their preferences regarding the coach 21 times. One of the nurses did not complete the settings for the coach. P3 and P5 changed the coach more often than the others (four and six times, respectively).

The key engagement metrics listed above are neatly organised below:

– Activities created per user: $\bar{x}=4.8$ for regular participants.
– Activity logs per activity: $\bar{x}=16$ for regular participants.
– Frequency of coach customisation: $\bar{x}=3$ per participant.
– Star updates: $\bar{x}=3.6$ per user; *total* = 29.
– Follow-up questions completed: $\bar{x}=8$ per participant.

While inferential statistical analysis was not conducted due to the sample size, the results indicate varied but sustained engagement over the course of the onboarding period and subjective experiences of changes in behaviour (Table 2), which in some also manifested changes in their star profile. Future studies with larger cohorts will use inferential statistics to assess the significance of changes in usage, long-term adherence, and behavioural outcomes.

**Table 2:** Overview of participants, their experience of VIP (number of health check-ups, or time worked if a nurse), changes in the star and/or related advice after using the app, subjective assessment of change due to use of the app, and the domain of the change. Readiness to change was assessed based on the three levels defined in Section 4.4.3: Low, Medium, High.

Participant	Gender	Age	VIP	Star^a^	Subj^a^	Domain	Transtheoretical model	Other apps
P1	F	63	3	No	Yes	Phys	High	No
P2	M	48	1	Yes	Yes	Nutr	High	No
P3	F	48	1	Yes	Yes	Nutr	Medium	Nutr
P4	F	36	0	Yes	Yes	Balance	Medium	No
P5	F	28	0	No	Yes	Phys	Medium	No
N1	F	31	1 year					Fitbit
N2	F	37	1 year					No
N3	F	N/A	1 month					Garmin

^a^ Descriptive results regarding behaviour change during the study period.

### 5.5 Summary of findings

The following is a summary of the findings relating to the five research questions in Section 3.4.

*RQ1 Facilitating engagement:* Engagement in the StarCoach application changed during the first weeks. The participants starting out with familiar, internalised activities facilitated the development of engagement. Making the star all green was viewed as the overall goal, which made them all update the star to see if there were changes. Some frustration was experienced when they were not prepared to change their behaviour to improve the star.

*RQ2 Ways of engagement:* There were different ways in which the participants engaged in the app, specifically, how the coach was utilised. The two participants who were assessed to be high in readiness to change, as defined in Section 4.4.3, did not (need or want to) elaborate on the pros and cons as they were already aware of why they should change. One of them did not like the agent telling them what to do, and instead wanted it to praise them even more when they were doing well. These two matched the ideal type 1 (35), i.e., people who are in the action stage of the TTM.

The participants who were assessed to be a medium level discussed why they need to change or why not, or that they did not want to change, during the sessions. These three participants can be described as resembling the ideal type 2, as they knew that it would be good for them if changes were made, but other things in life, in that moment, were considered of higher importance (35).

*RQ3 Use of BCTs* (8): The different modules implementing BCTs were used in the onboarding phase to different extents. Comments regarding what participants would like to have more of in the application related to the following BCTs: feedback on behaviour (2.2); feedback on outcomes of behaviour (2.7); reward (10.10); focus on past success (15.3); elevate challenging behaviour and a stronger focus on commitment (1.6); discrepancy between current behaviour and goal (1.9); and verbal persuasion about capability (15.1).

*RQ4 Coaching when goals are conflicting:* The participants managed their conflicting goals in the initial phase during their selection of activities. Further, they sometimes told the coach about their reasons for not conducting the activities when it asked. To what extent the coach was of help in this is unclear. However, one aspect relating to this was expressed by two participants. They indicated that they would like the coach to exhibit a stronger challenging attitude that would push them more to adhere to their plans.

*RQ5 Transparency and explainability:* The participants were able to explain and make sense of the system’s reasoning and decision-making related to the suggestions the coach delivered.

## 6 Related work

There is a multitude of health and fitness applications developed for particular purposes, such as increasing physical exercise, improving nutrition and sleep habits, and monitoring and reducing alcohol and tobacco use. They can be categorised as recommender systems, behaviour change systems, and persuasive technology, and some may take ethical considerations into account relating to being transparent, not deceitful, and only promoting and supporting user intention and desire to change behaviour (5, 44–46). The presented application targeting multiple lifestyle domains can be categorised as such an application, as the ethical aspects have been thoroughly explored throughout the participatory design process (17).

While there is a multitude of health-promoting applications targeting particular domains, there are only a few applications that adopt a more holistic perspective by taking multiple domains of interest into account, and these typically function by importing and sharing data with other health applications with a specific focus for a particular purpose. As an example, the application “Health Mate,” provided by Withings (France),^6^ has a holistic objective, knitting together parameters monitored by the user’s devices, e.g., pulse, ECG, blood pressure, weight, and sleep data. Another application is Life Cycle (Sleep Cycle AB, Sweden),^7^ a complementary application to the Sleep Cycle application, aimed at logging daily activities to see how these affect sleep.

Smartphone production companies have developed their own health applications, such as Apple’s Health and Fitness applications (US), which are included when buying an Apple smartphone, or Samsung’s Samsung Health, included when purchasing their smartphones or watches. Apple’s Health application targets a broad, holistic view on health, which is done by sharing personal data with third-party application providers to collect overviews of various health data, provided users install these applications and approve data sharing options.

Ethical aspects have been continuously explored from the initiation of the development of the StarCoach application (17). It was decided early in the process that the purpose of StarCoach is to be free to use to promote healthier lifestyles broadly across the population, which will, if successful, lead to decreased cost for society, besides gains in terms of improved health. This is also a result of VIP being available for free to all residents of the region between the ages of 40 and 60 (1).

Another significant difference of StarCoach is its holistic focus on *purposeful activity* in terms of activity theory, rather than solely on physical metrics such as steps, sleep, or pulse rate collected through bio-sensors. While fitness apps often categorise sensor data into specific sports, appealing to already active users, StarCoach targets individuals earlier in their readiness for change via daily activities. It outlines motives, barriers, and situated educative interventions to build sustainable habits. As these habits take hold, measurable outcomes may follow, generating effects measurable through bio-sensors as a consequence. Hence, a future update will add step counting to inform the star edge for physical activity.

## 7 Discussion

The StarCoach intervention and a user study were presented in this article. The particular focus for the user study was the first month of use, i.e., the onboarding phase, to explore the development of habits when using StarCoach. In the following sections, we discuss the findings along the following themes: attitudes towards health behaviour change; internalising new activities; behaviour change techniques, the mechanisms of action, and how they relate to purposes of behaviour change activity, translated into a framework for future studies; and finally, the methodology, strengths, and limitations.

### 7.1 Attitudes towards health behaviour change and internalising new activities

One main module of the application is the star profile, representing the user’s current health status while embedding the idea of a future health profile where the star would be filled with a green colour, the optimal state, if it is not already. It was described as “the better me” by one participant and was considered to be the most important part of the application.

An interesting aspect was the connection of the star to the everyday activities that the user defined, representing the small steps to take towards the green star. One participant expressed the commitment to doing the activities as arising from the act of defining and adding these to the list of activities to be done, which aligns with goal-setting theory (47). This commitment persisted even if they did not log activities for different reasons, as they said “I know that I have the activity in the app, this is enough.”

Another observation was the different levels of readiness to change and attitudes connected to their health behaviour activities. All the participants were aware of areas where there were expectations to behave differently, either from themselves or by their social environment or society. They expressed different levels of readiness to change, and in some cases, a non-interest in changing at that moment, with different motives for this. The multi-purpose approach taken to embed five different domains of health behaviour seemed to allow the participants to find at least one area they wanted to see changes in and could commit to scheduling an activity, while other areas were less relevant or interesting.

Research has shown that behaviour change is more successful if connecting a new behaviour to an existing behaviour (48). In our study, the participants internalised the use of the application by connecting the content of the application to the habits they already had. The application became relevant when they had something as a starting point to figure out how the application worked. This aligns with the activity-theoretical principles of mediating tools and object-orientedness (28): their internalised activity was used as a tool to try the app, which was the initial objective of the onboarding activity. Although the main purpose during the design process was to facilitate a focus on the formation of new habits, it seems valuable to at least initially begin with habituated activities to promote use and achievements in the first phase of use. This implies that the design of the tailored support may need to be further tailored to support transition from the initial phase.

### 7.2 BCTs, MoAs, and the purposes of behaviour change activity—a framework for future studies

When mapping StarCoach functionality to BCTs, 33 BCTs were identified across the three main modules (Table 1), primarily targeting MoAs such as readiness to change, motivation, self-efficacy, goal commitment, competence, and outcome expectations. Users can choose which functionalities and health behaviours to engage with. Future studies will explore how the application is used over time, which modules are preferred, and whether usage patterns shift. The presented study provides implications for future studies on BCTs. As some are more prominent in the application, we aim to explore BCTs *clustered by their behaviour change purposes*, linked with specific application functionalities:

1. Organisation of behaviour change activity: goal setting for behaviour and outcome, goal commitment, graded tasks (activity definition and star profile).
2. Engagement in behaviour change activity: habit formation, self-monitoring (visualisation of activity over time).
3. Engagement in learning about the effects of behaviour change activity: information about consequences, credible source (star advice and coach statements from an expert perspective).
4. Reflection on and engagement in positive effects and contradictions caused by the behaviour change activity: incompatible beliefs, pros and cons, verbal persuasion and rewards (coach agent).

The clustering of BCTs is based on the theoretical framework applied in the study and on the following findings.

#### 7.2.1 Organisation of behaviour change activity

The study provided results regarding the users’ organisation of behaviour change activity, based on how they addressed the goal-setting tasks, their reasoning about the definition of activity on different levels, and on how they increased the frequency. We would like to explore how participants in future studies choose to formulate baby-step activities that are on the level that they perceive as achievable. In a previous study, we found that the degree of specificity participants used when defining an activity mirrored their readiness level (38), which will be explored further in future studies.

#### 7.2.2 Engagement in behaviour change activity

As participants expressed different motives and ways to engage in activities, with social aspects prominent, we would like to explore to what extent participants define social activities, socially motivated activities, and activities that they assign companionship to for their execution. Further, we would like to follow up on the strategy observed in the presented study of beginning by adding activities they already do to explore whether this strategy leads to higher engagement over time.

#### 7.2.3 Engagement in learning about effects of behaviour change activity

The participants expressed to some extent that they already knew what they should do and why, which may be an effect of participating in VIP. We will study in what way participants in different age groups make use of the functionalities that make use of the domain knowledge base underpinning person-tailored advice and argument from expert opinion.

#### 7.2.4 Reflection on and engagement in positive effects and contradictions caused by the behaviour change activity

Some participants expressed a desire to see progress and changes in the star and receive some positive reinforcement when they are doing well. They also expressed frustration when the outcome was not as positive as they would wish, with a few wanting the coach to be more challenging. Cognitive dissonance in different situations and how participants may act on these would be interesting to study further. One example is the list of excuses, collected by the coach, which users will be able to revisit and reflect upon.

### 7.3 BCTs and MHBC in StarCoach

The StarCoach intervention aligns closely with principles of MHBC frameworks (3). It is a central idea in MHBC that health behaviours often cluster, and that modifying one may facilitate change in others. StarCoach uses this notion by supporting user-defined goals across multiple domains (e.g., physical activity, stress), and by embedding BCTs that target motivation, readiness for change, and self-efficacy across these areas simultaneously (8).

Unlike traditional MHBC interventions that often apply only a sequential change strategy such as the TTM (49), StarCoach adopts a personalised and concurrent strategy alongside a sequential strategy, allowing users to take the step to change when they feel ready. This has been shown to be, in some cases, superior to the sequential approach (50). This is aligned with MHBCs that emphasise stage-matched interventions (4), as implemented in our readiness to change model in Section 4.4.3. Furthermore, the adaptive and multi-modular design of StarCoach can aid in the scaffolding of motivation in a domain (e.g., physical activity) by supporting success in another (e.g., finding time to recover), which reflects the core approach of MHBC.

While MHBC frameworks provide a significant backbone for multi-behaviour interventions, StarCoach contributes a layer of computational adaptation and personalisation through its ontology-based engine (ACKTUS) and interactive coaching. Moreover, the coach’s adaptation to user-preferred coaching styles, barriers, and emotional responses potentially furthers engagement and adherence in comparison to static MHBC approaches that are tailored to the user’s behaviour stage.

### 7.4 Methodology and limitations

The participatory design process has elicited a wide range of aspects to consider, affecting the design and development of the StarCoach application. Besides the multidisciplinary perspective, the range of lifestyle domains covered, the age range in target groups, changes in the national guidelines, and new findings in research studies, there have also been organisational changes in healthcare and changes in regulations affecting AI-based applications. These contextual aspects add to the complexity and provide challenges; however, addressing these will also contribute to the ecological validity of the outcome.

The main limitation of the user study is that the participants knew their use of the application would be followed up within days and weeks, thus, they could be expected to adhere to using the application to a larger extent than those who download the application and use it without being contacted (10). In a future RCT study planned to be conducted through VIP, all the participants who consent will be contacted during their first 4 weeks of use after creating accounts, which is expected to increase adherence (14).

Another limitation is the small sample size. However, as the study aimed at qualitative exploration rather than definitive conclusions, the sample size was sufficient. The focus was on understanding the attitudes and behaviours to identify phenomena for future research. One such phenomenon concerns how participants with varying degrees of readiness to change engage with the application over time, the findings of which are reported in this article. The participants expressed diverse attitudes towards technology, and represented two of the three ideal types identified by Eriksson et al. (35), though none represented the low readiness group. This is typical in volunteer-based studies, where participants are usually more motivated than the general population. That said, this motivation enabled in-depth, longitudinal observation over 4 weeks. Future studies will recruit through VIP, increasing the likelihood of including participants with lower readiness to change.

It will be particularly interesting to further explore how users use activities they already conduct to promote adherence to the use of the application and facilitate the formation of new habits. We will also further study how the coach could balance being challenging and supporting, and exhibit more social behaviour to meet users’ expectations.

Finally, although statistical generalisations cannot be made from the findings due to a limited sample size, valuable insights and observations in this study can inform future studies. In particular, these include the framework presented in Section 8 and the versions of the application and the coach.

## 8 Conclusions and future work

The contributions of research presented in this article are three-fold: (i) the StarCoach, a multicomponent lifestyle intervention for health behaviour change to reduce the risk of cardiovascular diseases; (ii) a framework for studying multicomponent lifestyle interventions with multiple BCTs; and (iii) the qualitative results of the participants’ experiences and use of StarCoach in the onboarding phase.

The design of StarCoach is motivated by the following: (i) theories on human activity, motivation, and behaviour change; (ii) professional perspectives and experiences of health behaviour interventions in clinical practice; (iii) professional perspectives on citizens as members of a social context influencing health behaviour change; (iv) empirical studies of use in the onboarding phase, by health experts and general users; and (iv) design principles for human-centred artificial intelligence (HCAI) and BCSs.

Since StarCoach, as a result of the participatory design process, also embeds a substantial number of BCTs, a framework for the evaluation of clusters of BCTs was presented. The framework will be applied and evaluated in future studies on StarCoach and other behaviour change applications.

The qualitative study of the onboarding phase provided findings relating to facilitating engagement, ways of engagement, use of BCTs, coaching when goals are conflicting, and transparency and explainability. Our findings include, for instance, the observation that users may use already habituated activities to establish a routine to use the intervention. This will be implemented as a guiding strategy in StarCoach and the effects on adherence/retention and engagement will be studied in future work. Further, the participants reported increased engagement in their chosen lifestyle-change activities during the study period. These findings will be followed up in future studies to evaluate the effects on behaviour and attitudes towards lifestyle changes over a longer period of time.

The integration of digital interventions such as StarCoach, developed by a multidisciplinary team, into healthcare programs similar to VIP has the potential to broaden the reach of intervention programs and their impact. Such applications are scalable, personalised tools that can be used for tailored long-term healthy behaviour change support. After further trials, StarCoach could serve as a cost-effective and accessible resource for preventive health management, empowering users to make healthy decisions and take ownership of their health.

## Data Availability

The datasets presented in this article are not readily available due to privacy and ethical restrictions. Requests to access the datasets should be directed to helena.lindgren@umu.se.

## Footnotes

^1^ https://www.w3.org/OWL/

^2^ https://www.w3.org/RDF/

^3^ https://rdf4j.org/

^4^ https://www.who.int/standards/classifications/international-classification-of-functioning-disability-and-health

^5^ https://www.visual-paradigm.com/guide/development/what-is-rest-api/

^6^ https://www.withings.com/eu/en/

^7^ https://www.sleepcycle.com/

## Author's note

The article presents the StarCoach application and its development, which is not yet available to the general audience.
